# Prognostic Value of Albumin-to-CEA Ratio in Metastatic Colorectal Cancer: A Retrospective Study

**DOI:** 10.3390/biomedicines14030579

**Published:** 2026-03-05

**Authors:** Zekeriya Hannarici, Aykut Turhan, Mehmet Emin Buyukbayram, Alperen Akansel Çağlar, Mehmet Bilici, Salim Başol Tekin, Senar Ebinç, Ali Yılmaz, Birol Ocak, Pınar Çoban Eşdur, Salih Gölcüklü, Elif Bayraktar

**Affiliations:** 1 Department of Medical Oncology, Bursa City Education and Research Hospital, University of Health Sciences, Bursa 16250, Turkey; 2 Department of Medical Oncology, Ordu University Training and Research Hospital, Ordu 52200, Turkey; 3 Department of Medical Oncology, Yalova Training and Research Hospital, Yalova 77200, Turkey; 4 Department of Medical Oncology, Istanbul Çam and Sakura City Hospital, Istanbul 34480, Turkey; 5 Department of Medical Oncology, Atatürk University Faculty of Medicine, Erzurum 25240, Turkey; 6 Department of Medical Oncology, Yuzuncu Yil University Faculty of Medicine, Van 65080, Turkey; 7 Department of Medical Oncology, Nev Hospital, Şanlıurfa 63100, Turkey; 8 Department of Medical Oncology, Bursa Yüksek Ihtisas Training and Research Hospital, Bursa 16310, Turkey

**Keywords:** metastatic colorectal cancer, albumin-to-CEA ratio, prognostic biomarkers, progression-free survival, overall survival

## Abstract

**Background:** Finding dependable prognostic biomarkers for metastatic colorectal cancer (mCRC) is crucial. The albumin-to-carcinoembryonic antigen (CEA) ratio (ACR), a measure of nutritional-inflammatory status and tumor load, has emerged as a promising prognostic indicator. This study assessed ACR’s prognostic value of ACR in patients with mCRC. **Methods:** This retrospective study included 125 patients with mCRC followed at our institution between July 2010 and March 2022. ROC curve analysis was used to determine the optimal cutoff values for ACR, prognostic nutritional index (PNI), lymphocyte-to-monocyte ratio (LMR), and CEA. Kaplan–Meier and Cox regression analyses were used to evaluate progression-free survival (PFS) and overall survival (OS). **Results:** PFS and OS were 13.3 and 26.0 months, respectively. Patients with an ACR ≥ 4.24 experienced significantly longer PFS (16.8 vs. 11.0 months; *p* = 0.001) and OS (32.0 vs. 22.3 months; *p* < 0.001) compared with those with ACR < 4.24. In univariable analyses, ACR was significantly associated with both PFS and OS, whereas PNI, LMR, and CEA were associated with OS only. In multivariable Cox regression models ACR showed a significant association with both PFS (HR 0.413; 95% CI: 0.265–0.643; *p* < 0.001) and OS (HR 0.341; 95% CI: 0.210–0.551; *p* < 0.001), while maintenance therapy was significantly associated with PFS only and ECOG performance status, LMR and PNI with OS only. **Conclusions:** ACR appears to be a cost-effective biomarker that is associated with PFS and OS in mCRC. These findings suggest that ACR may have potential value for prognostic assessment and risk stratification in patients with mCRC.

## 1. Introduction

Metastatic colorectal cancer (mCRC) is a major global health issue characterized by a heavy disease burden and high mortality rate [1]. Although it ranks as the second leading cause of cancer-related deaths, advancements in treatment, such as chemotherapy, targeted therapy, and multidisciplinary strategies, have enhanced patient survival [1,2]. In recent times, the advent of targeted therapies and the development of new treatment approaches have further transformed the therapeutic landscape of mCRC [3]. Nevertheless, many individuals with stage IV mCRC continue to experience poor survival rates, with only approximately 14% surviving for five years [4]. Conventional prognostic factors, such as TNM staging and clinicopathological characteristics, often fall short in accurately predicting individual risk due to the heterogeneity of the disease [5]. This highlights the critical need for new, straightforward, and dependable prognostic biomarkers for mCRC [5,6].

There is a growing acknowledgment of the significant role that systemic inflammation and nutritional status play in cancer progression, including colorectal cancer [7,8]. The interplay between the immune-nutritional environment, disease severity, and patient survival is notable [9]. In recent years, although biomarkers such as the Prognostic Nutritional Index (PNI) and Lymphocyte-to-Monocyte Ratio (LMR) have demonstrated prognostic significance [9,10], the potential of combining tumor burden indicators, such as carcinoembryonic antigen (CEA), with nutritional markers, such as albumin, has not been thoroughly investigated [11,12]. There is a pressing need for easily accessible biomarkers that can integrate tumor biology with the host’s nutritional status to provide a more comprehensive prognostic evaluation [10].

Carcinoembryonic antigen is extensively used as a marker of tumor burden in the management of colorectal cancer [13,14]. It plays a vital role in tracking disease progression, metastasis, and treatment effectiveness [14,15]. High levels of CEA are associated with more aggressive disease, significant tumor burden, and lower survival rates [16,17,18]. Although its prognostic value is well established, CEA mainly reflects characteristics specific to the tumor and does not fully account for host-related factors, such as nutritional or systemic inflammatory status, which are also associated with prognosis [16].

Serum albumin, a protein produced by the liver, serves as an indicator of nutritional health, systemic inflammation, and physiological reserves in individuals with cancer [19,20]. Its importance as a prognostic marker in cancer, especially colorectal cancer, is increasingly being recognized. Low albumin levels or hypoalbuminemia are consistently associated with negative outcomes, such as poor postoperative recovery, decreased tolerance to chemotherapy, advanced disease stages, and reduced survival rates in various types of cancer [20,21]. Consequently, albumin is a significant yet often overlooked prognostic factor in mCRC, providing valuable insights into patients’ resilience against disease progression and treatment.

In line with this concept, previous studies, including a landmark report published in 2003, have demonstrated that serum albumin and CEA predict survival in patients with advanced colorectal cancer [22]. These findings established the prognostic relevance of host nutritional status and tumor burden in metastatic disease. However, these parameters have predominantly been evaluated in isolation. Whether integrating albumin and CEA into a single composite index provides additional prognostic information in mCRC remains insufficiently explored.

The albumin-to-CEA ratio (ACR) serves as a promising prognostic tool by simultaneously indicating the host’s nutritional inflammatory status and tumor burden. Although the ACR has demonstrated promising prognostic value in other cancers, such as gastric and rectal cancers [23,24], its specific prognostic role in mCRC remains largely unclear and under-researched. This underscores a significant gap in the knowledge of mCRC prognostication. This study sought to thoroughly assess the prognostic significance of ACR in patients with mCRC to enhance risk stratification and guide clinical decision-making. To the best of our knowledge, this is the first study to evaluate the prognostic value of the ACR specifically in patients with mCRC.

## 2. Material and Method

### 2.1. Subjects

From July 2010 to March 2022, 125 patients with mCRC were observed at our hospital’s oncology clinic and retrospectively included in this study. Given the long study period and the evolving landscape of systemic therapies, diagnostic approaches, and supportive care in mCRC, the calendar year of diagnosis was used as a surrogate for treatment era. Patients were categorized into two periods: 2010–2015 and 2016–2022. All procedures complied with the Declaration of Helsinki, and approval was obtained from the Institutional Ethics Committee. Data on patient demographics—including age, sex, smoking status, comorbidities, performance status, tumor location, metastatic sites, metastasectomy status, mutation profile, and microsatellite instability (MSI)—were retrieved from the hospital information management system. Information on chemotherapy regimens, biological agents, and maintenance treatment was obtained from electronic medical records. Molecular analyses were not consistently available throughout the study period and were primarily performed to guide treatment selection rather than for systematic prognostic assessment.

### 2.2. Exclusion Criteria

Patients were excluded if they had incomplete clinical or laboratory data, an active systemic infection, a chronic inflammatory condition, a history of another malignancy, a hematological disorder, or prior radiotherapy.

### 2.3. Laboratory Data

Laboratory data included hemoglobin (g/dL), red blood cell count (×10^6^/µL), red cell distribution width (%), leukocyte, neutrophil, lymphocyte, monocyte, and platelet counts (×10^3^/µL), glucose (mg/dL), aspartate aminotransferase (U/L), alanine aminotransferase (U/L), lactate dehydrogenase (U/L), albumin (g/dL), alkaline phosphatase (U/L), gamma-glutamyl transferase (U/L), creatinine (mg/dL), total bilirubin (mg/dL), and CEA (ng/mL). levels, were obtained from the hospital’s information management system. Complete blood count was performed using a Sysmex XN-9000 (Kobe, Japan) automated analyzer. For biochemical assessments, Beckman Coulter AU5821 (Brea, CA, USA) and Roche Cobas 8000 devices (Indianapolis, IN, USA) were used. The LMR value was calculated by dividing the lymphocyte count by the monocyte count; the PNI value was derived using the formula ‘(10 × albumin (g/dL) + (0.005 × total lymphocyte count)’; and the ACR value was determined by dividing 10 × albumin (g/dL) by the CEA (ng/mL).

### 2.4. Statistical Analysis

Continuous variables were presented as mean ± standard deviation (SD) or median with interquartile range (IQR), as appropriate. Overall survival (OS) was measured as the time span from the diagnosis of mCRC to either death or the most recent follow-up for those who were still alive. Progression-free survival (PFS) was defined as the interval from diagnosis to either disease advancement or death, or until the last follow-up for patients who showed no signs of disease progression. Kaplan–Meier curves were used to determine PFS and OS durations, which were then compared with categorical variables using the log-rank test. The receiver operating characteristic (ROC) curve was used to identify the optimal cutoff values for ACR, PNI, LMR, and CEA in terms of sensitivity and specificity.

ACR was calculated as serum albumin (g/dL) divided by serum CEA (ng/mL), using baseline laboratory measurements obtained prior to initiation of systemic therapy. No unit conversion was performed. In this cohort, no CEA values were below the assay detection limit; therefore, no constant addition or data imputation procedures were applied. In addition to cut-off–based analyses, ACR was also modelled as a continuous variable in multivariable Cox regression models to assess robustness and to avoid potential information loss due to dichotomisation. No log-transformation, truncation, or Winsorisation procedures were applied, as no biologically implausible extreme values were observed and results remained stable in continuous modelling.

The Chi-square (X2) test was used to evaluate the relationship between the groups.

To evaluate the prognostic significance of variables such as age, sex, smoking status, pre-existing health conditions, performance status, tumor location, metastasis site, metastasectomy, mutation status, MSI results, initial chemotherapy regimens, use of first-line biological agents, maintenance therapy, ACR, PNI, LMR, and CEA on PFS and OS, both univariate and multivariate Cox regression analyses were performed. To minimize potential confounding related to surgical selection, additional subgroup analyses were performed after excluding patients who underwent metastasectomy. This approach was used to calculate HRs and 95% CIs. All statistical analyses were performed using SPSS version 21, with statistical significance defined as *p* < 0.05.

## 3. Results

A total of 125 patients were included in the study. Forty-two patients (33.6%) were diagnosed between 2010 and 2015, while 83 patients (66.4%) were diagnosed between 2016 and 2022. The cohort consisted of 58 females (46.4%) and 67 males (53.6%). The median age was 57 years (range, 19–86), and 84.8% (*n* = 106) of patients had an ECOG performance status < 2. The majority of tumors were located in the left colon and rectum (81.6%). Liver metastasis was present in 44.8% (*n* = 56) of patients, and 31.2% (*n* = 39) had metastases at multiple sites. Metastasectomy was performed in 18.4% (*n* = 23) of cases. Most patients received combination chemotherapy, and maintenance treatment was applied in 39.2% (*n* = 49) of the cohort. During follow-up, 111 patients (88.8%) experienced disease progression, and 97 patients (77.6%) died.

The areas under the curves for ACR, PNI, LMR, and CEA were 0.69, 0.62, 0.63, and 0.68, respectively. The ideal cutoff points for PNI, LMR, and CEA, based on sensitivity and specificity, were determined to be 38, 2.91, and 8.98, respectively. For ACR, the most effective cutoff value was 4.24, with a sensitivity of 68% and specificity of 64%.

The median ACR in the overall cohort was 2.64 (IQR: 0.40–10.10). Based on predefined cut-off values, 56.8% of patients had an ACR < 4.24, 55.2% had a PNI < 38, 56.6% had an LMR < 2.91, and 42.4% had a CEA ≥ 8.98. Detailed demographic, clinicopathological, treatment, and biomarker characteristics are presented in Table 1.

Significant associations were observed between LMR and age (*p* = 0.004), PNI and ECOG performance status (*p* = 0.024). In addition, ACR was associated with metastatic sites (*p* = 0.001), Treatment era (*p* = 0.009) and CEA levels (*p* < 0.005). Lower ACR values were more frequently observed in patients with liver metastases and in those with multiple metastatic sites, suggesting a relationship between reduced ACR and higher tumor burden. The relationships between inflammatory indices and clinicopathological variables are summarized in Table 2.

**Figure 1:**
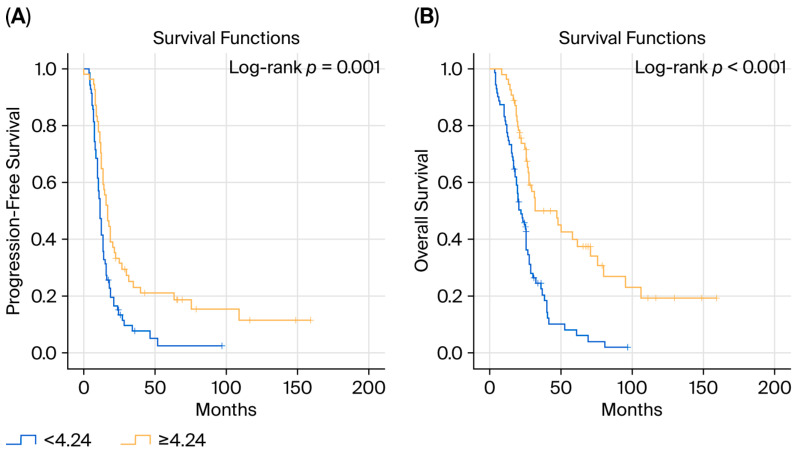
Kaplan–Meier curves for PFS (**A**) and OS (**B**) according to albumin-to-CEA ratio (ACR).

At the time of analysis, the median PFS was 13.3 months and the median OS was 26 months. Patients with an ECOG performance status < 2 had a significantly longer OS than those with ECOG ≥ 2 (27.8 vs. 13.3 months, *p* < 0.001), whereas PFS did not differ significantly (13.6 vs. 10.5 months, *p* > 0.05). Maintenance therapy was associated with a significantly longer PFS (16.8 vs. 11.0 months, *p* = 0.002), while the difference in OS was not statistically significant. With respect to nutritional and inflammatory indices, PFS did not differ significantly according to PNI or LMR categories; however, OS was significantly longer in patients with high PNI (27.9 vs. 25.6 months, *p* = 0.005) and high LMR (32.4 vs. 22.3 months, *p* = 0.002). Notably, patients with an ACR ≥ 4.24 demonstrated significantly longer PFS (16.8 vs. 11.0 months, *p* = 0.001) and OS (32.0 vs. 22.3 months, *p* < 0.001) compared with those with lower ACR values (Figure 1). The associations between categorical variables and survival outcomes are summarized in Table 3.

The associations between PFS, OS, clinicopathological parameters, and biomarkers were evaluated using Cox regression analyses (Table 4). Treatment era (2010–2015 vs. 2016–2022) was not significantly associated with either PFS or OS in univariable analyses. In univariable Cox regression analyses for PFS, maintenance treatment (HR 0.544; 95% CI: 0.368–0.803; *p* = 0.002), ACR (HR 0.528; 95% CI: 0.357–0.780; *p* = 0.001), and CEA (HR 1.799; 95% CI: 1.205–2.625; *p* = 0.003) were significantly associated with outcomes. For OS, univariable analyses demonstrated significant associations with ECOG performance status (HR 2.973; 95% CI: 1.718–5.148; *p* < 0.001), ACR (HR 0.384; 95% CI: 0.249–0.592; *p* < 0.001), PNI (HR 0.566; 95% CI: 0.366–0.843; *p* = 0.006), LMR (HR 0.522; 95% CI: 0.343–0.794; *p* = 0.002), and CEA (HR 2.455; 95% CI: 1.596–3.777; *p* < 0.001) (Table 4).

Clinically relevant variables, tumour-burden proxy variables (liver metastasis and number of metastatic sites), and variables showing significance in univariable analyses were considered in multivariable Cox regression models.

In the updated PFS model, maintenance treatment (HR 0.50; 95% CI: 0.33–0.74; *p* < 0.001) and ACR (HR 0.41; 95% CI: 0.27–0.64; *p* < 0.001) remained significantly associated with PFS after adjustment for age, ECOG performance status, and tumour-burden proxies.

In the OS model, ECOG performance status (HR 5.13; 95% CI: 2.61–10.09; *p* < 0.001), ACR (HR 0.34; 95% CI: 0.21–0.55; *p* < 0.001), LMR (HR 0.63; 95% CI: 0.41–0.98; *p* = 0.040), and PNI (HR 0.62; 95% CI: 0.39–0.97; *p* = 0.035) remained significantly associated with OS. Liver metastasis and number of metastatic sites were not significantly associated with survival outcomes (Table 5).

The Cox proportional hazards model demonstrated good discriminative ability for OS (Harrell’s C-index = 0.71) and moderate discriminative ability for PFS (C-index = 0.67).

In addition to the cut-off–based analysis, ACR was further evaluated as a continuous variable to avoid potential information loss due to dichotomisation. In the multivariable PFS model adjusting for age, ECOG performance status, liver metastasis, number of metastatic sites, and maintenance treatment, continuous ACR remained significantly associated with PFS (HR per unit increase: 0.95; 95% CI: 0.93–0.98; *p* < 0.001). Similarly, in the OS model adjusting for age, ECOG performance status, tumour-burden proxies, LMR, and PNI, continuous ACR remained significantly associated with OS (HR per unit increase: 0.94; 95% CI: 0.91–0.97; *p* < 0.001). These findings support the robustness of the association between ACR and survival outcomes beyond cut-off–based modelling.

To further address the potential incremental value of ACR beyond serum CEA levels, additional multivariable Cox models were constructed including continuous CEA values alongside tumour-burden proxies and inflammatory indices. In the updated PFS model, ACR remained significantly associated with PFS (HR 0.41; 95% CI: 0.26–0.65; *p* < 0.001), whereas continuous CEA was not significantly associated with PFS (*p* = 0.985). Similarly, in the OS model, ACR retained statistical significance (HR 0.36; 95% CI: 0.22–0.58; *p* < 0.001), while continuous CEA did not remain significantly associated with OS (*p* = 0.097). These findings suggest that the prognostic association of ACR is not solely explained by serum CEA levels.

In subgroup analyses limited to patients who did not undergo metastasectomy, ACR and maintenance treatment remained significantly associated with longer PFS (ACR: HR 0.430; 95% CI: 0.270–0.680; *p* < 0.001; maintenance treatment: HR 0.530; 95% CI: 0.340–0.820; *p* = 0.004), while ECOG performance status showed a borderline association. Similarly, in the OS analysis, ECOG performance status and ACR remained significantly associated with survival, with high ACR (≥4.24) being associated with a significantly reduced risk of death (HR 0.310; 95% CI: 0.180–0.520; *p* < 0.001). Importantly, other inflammation- and nutrition-related indices, including LMR and PNI, did not retain statistical significance in this restricted model.

## 4. Discussion

Survival outcomes in mCRC are influenced by a complex interplay of clinical, pathological, molecular, and host-related factors [1]. Although there have been significant advancements in treatment strategies that have improved patient survival, the diverse nature of mCRC requires a continuous search for new predictive and prognostic biomarkers to enhance risk stratification and tailor personalized treatment plans [5,6]. In univariable analyses, maintenance treatment, CEA, and ACR were associated with PFS, whereas ECOG performance status, CEA, PNI, LMR, and ACR were associated with OS. In multivariable analyses, ECOG performance status, LMR and PNI remained significantly associated with OS, while maintenance therapy was significantly associated with PFS. Importantly, ACR remained significantly associated with both PFS and OS after multivariable adjustment, distinguishing it from the other evaluated biomarkers. Notably, these associations persisted in sensitivity analyses excluding patients who underwent metastasectomy, with ACR continuing to show a consistent association with both PFS and OS, supporting the robustness of the observed findings.

In recent years, systemic inflammation has been increasingly acknowledged as a vital factor in the intricate processes of tumor development, metastasis, immune suppression, and resistance to treatment, playing a significant role in determining cancer outcomes [25,26,27,28]. The tumor microenvironment, characterized by persistent inflammation, actively encourages immunosuppression and tumor progression [29,30]. The LMR is an established inflammatory marker associated with survival in various cancers, including CRC [9,31,32]. In CRC, a low LMR is thought to indicate impaired lymphocyte-mediated antitumor immunity, together with increased monocyte-driven tumor-associated macrophage activity, both of which contribute to tumor progression and an unfavorable prognosis [33]. In our research, LMR was found to have a notable correlation with OS in both univariate and multivariable analyses, while no significant link was identified for PFS. This outcome indicates that although LMR might indicate inflammatory status, its prognostic value could be restricted to specific outcomes, such as OS, within our mCRC patient group.

The PNI, which combines serum albumin levels and lymphocyte counts, serves as an indicator of a patient’s immunonutritional condition and has been consistently linked to outcomes in gastrointestinal cancers [34,35,36]. A low PNI often signals poor nutritional health and immune system issues, which are associated with decreased survival rates and lower tolerance to cancer treatments in various types of cancer [37,38]. Many patients with cancer experience nutritional deficiencies [39]. Consistent with this, our study identified a link between PNI and OS in the univariate analysis and multivariable analyses. In line with this, our research found a connection between PNI and OS in both univariate and multivariable analyses. Nevertheless, akin to LMR, its prognostic link was confined to OS, as it did not show a significant correlation with PFS in our mCRC cohort. This indicates a more complex array of factors affecting the prognosis of mCRC. The enhanced prognostic capability of ACR might be attributed to its capacity to encompass both tumor burden and the host’s immuno-nutritional status, offering a more comprehensive depiction of disease biology compared to single-component markers like PNI, LMR, and CEA.

Malnutrition is a crucial prognostic factor in cancer, especially among patients with mCRC, who often suffer from cachexia, systemic inflammation, and treatment-related metabolic issues [40,41,42]. Serum albumin, an essential protein produced by the liver, acts as a broad indicator of nutritional health, has anti-inflammatory effects, possesses antioxidant properties, and indicates the overall synthetic function of the liver [20,43]. Numerous studies, including recent reports, have identified hypoalbuminemia, a sign of poor nutritional health, as a negative prognostic factor associated with reduced OS, more advanced disease, higher postoperative complications, and decreased chemotherapy tolerance in patients with colorectal cancer [20,21,44]. Although albumin plays a role in patient survival by indicating the body’s physiological reserve and inflammatory status, its standalone use may be limited, highlighting the potential advantage of integrating it with other markers into composite indices for improved prognostic accuracy. Hypoalbuminemia has consistently been associated with poor outcomes across multiple malignancies, reflecting cancer-related cachexia, systemic inflammation, and reduced physiological reserve rather than tumor-specific biological effects [45]. Similarly, elevations in CEA have been reported not only in colorectal cancer but also in gastric, pancreatic, lung, and breast malignancies, as well as in certain benign inflammatory conditions [46]. In healthy individuals, serum CEA levels are generally below 5 ng/mL, whereas patients with intestinal polyps typically demonstrate values within the normal range or only mild elevations [47]. Taken together, these observations suggest that, like albumin, CEA is not a tumor-specific marker but rather reflects broader disease- and host-related processes.

Nevertheless, CEA is widely used as a surrogate marker of tumor burden in colorectal cancer, reflecting disease activity, metastatic potential, and aggressive tumor biology, particularly in patients with extensive or liver-dominant metastatic involvement [13,14,48]. Consistently, elevated CEA levels have been associated with increased tumor burden and poorer survival outcomes across multiple studies [16,17,18], and CEA is routinely employed to monitor treatment response and disease progression due to its accessibility and cost-effectiveness [14,15]. In the present study, CEA was significantly associated with both PFS and OS in univariate analyses; however, it did not retain prognostic significance in multivariate models, consistent with previous reports which suggest that while CEA reflects tumor burden, it does not fully capture the complex interplay between tumor biology and host-related factors that ultimately influence survival. The prognostic relevance of serum albumin and CEA in advanced colorectal cancer has been well established for more than two decades, and our findings are therefore consistent with, rather than contradictory to, the existing literature [22,49]. Accordingly, ACR should be viewed not as a disease-specific biomarker for colorectal cancer, but as a composite indicator integrating tumor burden and biological aggressiveness, reflected by CEA levels, with the host’s nutritional and inflammatory status, reflected by serum albumin [24,50]. Importantly, ACR should not be interpreted as a disease-specific biomarker for colorectal cancer; rather, it represents a composite indicator of tumor burden and host condition, a biological interaction that is relevant across different malignancies [23,51]. Moreover, to minimize the potential confounding effects of treatment-related toxicity, ACR was assessed at baseline, prior to the initiation of systemic therapy, supporting its role as a marker of disease burden and host reserve rather than chemotherapy-induced effects [24,50,52]. Through the integration of these complementary dimensions, ACR may provide a more comprehensive prognostic assessment than either parameter alone and may reflect a biological interaction that appears to be relevant across different cancer types. Supporting this concept, Li et al. demonstrated that lower ACR values were significantly associated with poorer survival outcomes in resectable gastric cancer, underscoring the potential prognostic value of integrating nutritional status with tumor burden even in localized disease [23]. Similarly, in rectal cancer, Xie et al. reported that decreased ACR was significantly associated with higher recurrence rates and reduced OS, underscoring its utility as an effective tool for prognostic stratification [24]. Collectively, these findings suggest that the prognostic value of ACR arises from its ability to simultaneously capture tumor-related biological behavior and the host’s systemic response, providing an integrated measure of disease severity that may outperform single-component biomarkers.

In addition, given the strong prognostic impact of metastasectomy, we performed a restricted analysis limited to non-resected patients to minimize surgical selection bias. The persistence of ACR as a significantly prognostic factor for both OS and PFS in this uniformly palliative cohort indicates that its prognostic value is not driven by surgically treated long-term survivors but reflects underlying tumor burden and host-related biological characteristics.

Considering the extended duration of the study, the link observed between ACR and the treatment periods (2010–2015 versus 2016–2022) is not surprising. This result likely indicates shifts in clinical practices over time rather than a direct biological impact, as previously noted in studies highlighting the evolution of treatment strategies for mCRC [53,54]. Over the years, advancements in systemic therapies, such as the introduction of more effective drugs and combination treatments, along with enhancements in supportive care, may have affected inflammatory and nutritional status, as indicated by ACR [55,56]. Additionally, changes in patient selection, including earlier diagnoses and better baseline performance status in recent years, might have also played a role in this association [57].

Despite this significant association, the era of treatment did not show a correlation with OS or PFS in Kaplan–Meier, univariate, or multivariate analyses. This suggests that although there is a connection between ACR and the treatment era, it might not have a direct impact on prognosis. Instead, it probably reflects changes in treatment strategies or patient characteristics over time, consistent with previous findings [53].

Nevertheless, even with this notable connection, the treatment era did not correlate with OS or PFS in Kaplan–Meier, univariate, or multivariate analyses. This indicates that while there is a link between ACR and the treatment era, it may not directly affect prognosis. Instead, it likely mirrors variations in treatment approaches or patient characteristics over time, aligning with earlier findings [58].

Our results are consistent with and build upon the findings of other cancers, clearly showing that an elevated ACR is strongly linked to prolonged PFS and OS in mCRC. Importantly, this study provides evidence supporting ACR as a complementary and easily obtainable prognostic marker reflecting host–tumor interaction, rather than a cancer-specific indicator.

The ease, affordability, and widespread availability of albumin and CEA measurements make the ACR an appealing and easily applicable tool for risk assessment in routine clinical settings. These findings highlight the potential of the ACR to provide valuable insights beyond traditional prognostic factors. Nonetheless, the retrospective design of this study calls for further validation through prospective multicenter clinical trials to confirm these results and establish the role of ACR in guiding treatment decisions for patients with mCRC.

## 5. Limitations

This study has several limitations that should be acknowledged. First, its retrospective and single-center design inherently limits the generalizability of the findings. Second, comprehensive molecular profiling—including detailed RAS/RAF subclonal variants, MSI subtypes, and tumor mutational burden—was not consistently available throughout the study period, reflecting real-world clinical practice in earlier years. Although these molecular features are known to influence prognosis and treatment selection in mCRC, substantial missing data precluded their reliable inclusion in multivariable analyses; therefore, residual confounding related to unmeasured molecular variables cannot be excluded. Accordingly, the prognostic value of ACR should be interpreted as complementary rather than substitutive to established molecular biomarkers and warrants further validation in molecularly annotated cohorts. Third, dynamic changes in serum albumin and CEA levels during treatment were not evaluated, preventing assessment of the potential prognostic value of longitudinal ACR measurements. In addition, factors that may influence albumin levels—such as nutritional interventions, systemic inflammation, liver function, and comorbid conditions—were not uniformly documented and may have affected ACR values. Finally, due to the retrospective nature of the study, chemotherapy-related adverse events and patient-reported symptoms (including fatigue, anorexia, weight loss, and gastrointestinal complaints) were not systematically or uniformly recorded in medical records. Adverse events were not consistently graded according to CTCAE criteria, and symptom burden could therefore not be reliably analyzed. Although the sample size was adequate for the primary analyses, external validation in larger, prospective, multicenter cohorts is required to confirm the prognostic significance of ACR in mCRC.

## 6. Conclusions

In summary, this study suggests that the ACR represents a simple, cost-effective, and readily available biomarker that is significantly associated with both PFS and OS in patients with mCRC. While conventional markers such as CEA, PNI, and LMR showed prognostic associations in univariate analyses, ACR was the only biomarker that remained significantly associated with outcomes after multivariable adjustment, highlighting its ability to capture information related to both tumor burden and host nutritional–inflammatory status. Given its practicality and consistent association with survival outcomes, ACR may offer added value for prognostic stratification and risk assessment in routine clinical practice. Nevertheless, these findings should be interpreted within the context of a retrospective single-center design. Prospective, multicenter studies with larger cohorts are warranted to validate these results and to further clarify the potential role of ACR within personalized treatment strategies for mCRC.

## Figures and Tables

**Table 1:** Demographic and clinicopathological data of the patients.

Variable	*n*	%
**Gender**		
Female	58	46.4
Male	67	53.6
**Age (years)**		
<57	59	47.2
≥57	66	52.8
**ECOG performance status**		
<2	106	84.8
≥2	19	15.2
**Smoking status**		
Smoker	47	37.6
Non-smoker	78	62.4
**Comorbidities**		
None	77	61.6
CAD	29	23.2
DM	9	7.2
Other	10	8.0
**Primary tumor location**		
Right colon	18	14.4
Left colon	65	52.0
Transverse colon	5	4.0
Rectum	37	29.6
**Metastatic site**		
Lymph node	4	3.2
Peritoneum	13	10.4
Liver	56	44.8
Local invasion	13	10.4
Multiple sites	39	31.2
**Metastasectomy**		
No	102	81.6
Yes	23	18.4
**Mutation status**		
RAS wild-type	54	43.2
RAS mutant	45	36.0
BRAF mutant	2	1.6
Not available	24	19.2
**MSI status**		
MSS	23	18.4
MSI-high	3	2.4
Not available	99	79.2
**First-line chemotherapy**		
Oxaliplatin-based regimen	77	61.6
Irinotecan-based regimen	43	34.4
Single-agent regime	5	4.0
**First-line biological agent**		
Anti-VEGF	74	59.2
Anti-EGFR	22	17.6
None	29	23.2
**Maintenance therapy**		
No	76	60.8
Yes	49	39.2
**Treatment era**		
2010-2015	42	33.6
2016-2022	83	66.4
**Disease progression**		
No	14	11.2
Yes	111	88.8
**Patient’s survival status**		
Alive	28	22.4
Death	97	77.6
**ACR**		
<4.24	71	56.8
≥4.24	54	43.2
**PNI**		
<38	69	55.2
≥38	56	44.8
**LMR**		
<2.91	72	56.6
≥2.91	53	43.4
**CEA**		
<8.98	54	57.6
≥8.98	71	42.4

ACR: albumin-to-CEA ratio; CAD: Coronary artery disease; CEA: Carcinoembryonic antigen; DM: diabetes mellitus; ECOG: Eastern Cooperative Oncology Group; LMR: Lymphocyte-to-Monocyte Ratio; MSI: microsatellite instability; MSS: microsatellite stabil; PNI: prognostic nutritional index.

**Table 2:** Comparison of categorical data with LMR, PNI, and ACR.

Variable	*n*	LMR < 2.91	LMR ≥ 2.91	*p*	PNI < 38	PNI ≥ 38	*p*	ACR < 4.24	ACR ≥ 4.24	*p*
**Gender**										
Female	58	30	28		32	26		35	23	
Male	67	42	25	0.216	37	30	0.995	36	31	0.457
**Age (years)**										
<57	59	26	33		28	31		37	22	
≥57	66	46	20	**0.004**	41	25	0.100	34	32	0.207
**ECOG performance status**										
<2	106	60	46		54	52		61	45	
≥2	19	12	7	0.594	15	4	**0.024**	10	9	0.690
**Smoking status**										
Smoker	47	27	20		27	20		25	22	
Non-smoker	78	45	33	0.979	42	36	0.695	46	32	0.527
**Comorbidities**										
None	77	40	37		38	39		47	30	
CAD	29	19	10		19	10		15	14	
DM	9	7	2		6	3		6	3	
Other	10	6	4	0.368	6	4	0.692	3	7	0.261
**Primary tumor site**										
Right colon	18	15	3		11	7		11	7	
Left colon	65	36	29		37	28		32	33	
Transverse colon	5	2	3		3	2		4	1	
Rectum	37	19	18	0.083	18	19	0.808	24	13	0.325
**Metastatic site**										
Lymph node	4	2	2		1	3		2	2	
Peritoneum	13	9	4		9	4		3	10	
Liver	56	31	25		32	24		34	22	
Local invasion	13	7	6		7	6		3	10	
Multiple sites	39	23	16	0.913	20	19	0.614	29	10	**0.001**
**Metastasectomy**										
No	102	57	45		57	45		61	41	
Yes	23	15	8	0.413	12	11	0.747	10	13	0.153
**Treatment era**										
2010–2015	42	24	18		21	21		17	25	
2016–2022	83	48	35	0.941	48	35	0.406	54	29	**0.009**
**CEA**										
<8.98	54	30	24		30	24		1	53	
≥8.98	71	42	29	0.687	39	32	0.944	70	1	**<0.005**

ACR: albumin-to-CEA ratio; CAD: Coronary artery disease; CEA: Carcinoembryonic antigen; DM: diabetes mellitus; ECOG: Eastern Cooperative Oncology Group; LMR: Lymphocyte-to-Monocyte Ratio; MSI: microsatellite instability; PNI: prognostic nutritional index.

**Table 3:** Relationship between categorical data and PFS and OS.

Variable	PFS (Months) Mean	Median	*p*	OS (Months) Mean	Median	*p*
**Gender**						
Female	27.7	13.5		41.4	27.9	
Male	27.4	12.6	0.894	43.7	25.7	0.842
**Age (years)**						
<57	28.6	12.4		45.0	27.8	
≥57	25.8	13.7	0.912	41.4	24.8	0.462
**ECOG performance status**						
<2	28.7	13.6		47.2	27.8	
≥2	16.4	10.5	0.193	18.4	13.3	**<0.001**
**Smoking status**						
Smoker	33.7	13.7		50.5	29.6	
Non-smoker	24.3	12.6	0.179	38.2	28.8	0.153
**Comorbidities**						
None	27.5	14.1		45.3	26.8	
CAD	22.1	13.3		36.8	25.7	
DM	11.9	11.1		20.9	16.0	
Other	38.3	10.2	0.404	51.7	32.0	0.199
**Primary tumor site**						
Right colon	12.1	12.6		26.7	20.1	
Left colon	28.1	13.7		46.4	26.8	
Transverse colon	14.7	11.0		30.7	25.7	
Rectum	35.2	12.3	0.090	50.5	28.8	0.095
**Metastatic site**						
Lymph node	12.9	7.9		31.4	22.3	
Peritoneum	19.9	15.6		38.5	27.4	
Liver	26.9	13.7		39.8	26.5	
Local invasion	40.9	14.5		74.0	31.9	
Multiple sites	21.5	10.2	0.461	31.7	23.2	0.141
**Metastasectomy**						
No	27.5	13.3		42.2	25.7	
Yes	23.4	14.5	0.883	45.0	36.4	0.290
**Treatment era**						
2010–2015	30.5	12.6		47.9	25.7	
2015–2022	20.9	13.5	0.789	34.6	27.6	0.632
**Maintenance therapy**						
No	21.2	11.0		40.5	24.6	
Yes	36.1	16.8	**0.002**	49.0	31.9	0.104
**ACR**						
<4.24	16.5	11.0		26.3	22.3	
≥4.24	39.0	16.8	**0.001**	64.1	32.0	**<0.001**
**PNI**						
<38	17.7	13.4		31.2	25.6	
≥38	39.7	12.6	0.097	60.5	27.9	**0.005**
**LMR**						
<2.91	22.6	13.3		34.3	22.3	
≥2.91	34.4	13.5	0.071	55.9	32.4	**0.002**
**CEA**						
<8.98	37.6	15.9		63.0	32.0	
≥8.98	16.9	11.0	**0.003**	26.7	23.0	**<0.001**

ACR: albumin-to-CEA ratio; CAD: Coronary artery disease; CEA: Carcinoembryonic antigen; DM: diabetes mellitus; ECOG: Eastern Cooperative Oncology Group; LMR: Lymphocyte-to-Monocyte Ratio; OS: Overall survival; PFS: Progression-free survival; PNI: Prognostic nutritional index.

**Table 4:** Univariable Cox regression analysis for PFS and OS.

Variable	PFS HR (95% CI)	*p* Value	OS HR (95% CI)	*p* Value
Gender	0.975 (0.668–1.422)	0.894	0.960 (0.640–1.440)	0.842
Age	1.021 (0.702–1.487)	0.912	1.162 (0.778–1.734)	0.463
ECOG performance status	1.408 (0.838–2.365)	0.196	2.973 (1.718–5.148)	**<0.001**
Cigarette smoking	1.309 (0.882–1.943)	0.181	1.357 (0.891–2.068)	0.155
Comorbid disease	1.034 (0.847–1.261)	0.744	1.028 (0.842–1.254)	0.790
Tumor site	0.861 (0.713–1.041)	0.123	0.852 (0.698–1.039)	0.113
Metastasis site	1.022 (0.960–1.089)	0.492	0.999 (0.933–1.070)	0.975
Liver metastasis	0.847 (0.580–1.238)	0.392	0.960 (0.638–1.445)	0.846
Multiple metastasis	1.337 (0.894–1.999)	0.157	1.447 (0.944–2.217)	0.090
Metastasectomy	0.964 (0.593–1.567)	0.883	0.754 (0.446–1.275)	0.292
Mutation analysis	0.881 (0.772–1.004)	0.058	0.960 (0.839–1.099)	0.557
MSI status	0.927 (0.791–1.086)	0.348	1.033 (0.857–1.246)	0.734
Treatment era (2016–2022 vs. ≤2015)	1.056 (0.708–1.575)	0.789	1.111 (0.721–1.712)	0.632
First-line CT regimens	0.974 (0.708–1.341)	0.872	1.153 (0.816–1.628)	0.420
First-line biological agent	0.970 (0.828–1.137)	0.709	0.936 (0.788–1.112)	0.454
Maintenance treatment	**0.544 (0.368–0.803)**	**0.002**	0.714 (0.474–1.074)	0.106
ACR (<4.24 vs. ≥4.24)	**0.528 (0.357–0.780)**	**0.001**	**0.384 (0.249–0.592)**	**<0.001**
PNI (<38 vs. ≥38)	0.722 (0.490–1.063)	0.099	**0.566 (0.366–0.843)**	**0.006**
LMR (<2.91 vs. ≥2.91)	0.703 (0.479–1.033)	0.073	**0.522 (0.343–0.794)**	**0.002**
CEA (<8.98 vs. ≥8.98)	**1.799 (1.205–2.625)**	**0.004**	**2.455 (1.596–3.777)**	**<0.001**

ACR: albumin-to-CEA ratio; CEA: Carcinoembryonic antigen; CT: chemotherapy; ECOG: Eastern Cooperative Oncology Group; LMR: Lymphocyte-to-Monocyte Ratio; MSI: microsatellite instability; OS: Overall survival; PFS: Progression-free survival; PNI: Prognostic nutritional index.

**Table 5:** Multivariable Cox regression analysis for overall PFS and OS.

Variable	PFS HR (95% CI)	*p* Value	OS HR (95% CI)	*p* Value
Age	0.999 (0.983–1.014)	0.859	0.991 (0.974–1.009)	0.350
ECOG performance status	1.700 (0.941–3.072)	0.079	5.131 (2.611–10.085)	**<0.001**
Liver metastasis	0.725 (0.437–1.203)	0.213	0.904 (0.516–1.586)	0.726
Multiple metastatic sites	0.729 (0.416–1.275)	0.268	1.122 (0.619–2.033)	0.704
Maintenance treatment	0.495 (0.329–0.744)	**<0.001**	—	—
ACR (<4.24 vs. ≥4.24)	0.413 (0.265–0.643)	**<0.001**	0.341 (0.210–0.551)	**<0.001**
LMR (<2.91 vs. ≥2.91)	—	—	0.632 (0.408–0.979)	**0.040**
PNI (<38 vs. ≥38)	—	—	0.616 (0.392–0.967)	**0.035**

ACR: albumin-to-CEA ratio; ECOG: Eastern Cooperative Oncology Group; LMR: Lymphocyte-to-Monocyte Ratio; OS: Overall survival; PFS: Progression-free survival; PNI: Prognostic nutritional index.

## Data Availability

The datasets generated and/or analyzed during the current study are not publicly available because of patient privacy and institutional data protection policies but are available from the corresponding author upon reasonable requests.

## Abbreviations

The following abbreviations are used in this manuscript:

ACR	albumin-to-CEA ratio
CAD	Coronary artery disease
CEA	Carcinoembryonic antigen
CT	chemotherapy
DM	diabetes mellitus
ECOG	Eastern Cooperative Oncology Group
LDH	lactate dehydrogenase
LMR	Lymphocyte-to-Monocyte Ratio
mCRC	metastatic colorectal cancer
MSI	microsatellite instability
MSS	microsatellite stabil
OS	overall survival
PFS	progression-free survival
PNI	prognostic nutritional index
RDW	red cell distribution width
ROC	receiver operating characteristic
